# Anatomical-Foliar Diversity of *Agave salmiana* subsp. *salmiana* (Asparagaceae) in Three Populations of the Teotihuacán Region (Mexico)

**DOI:** 10.3390/plants13223195

**Published:** 2024-11-14

**Authors:** Estela Sandoval-Zapotitla, Lorena E. Chávez-Güitrón, Florencia del C. Salinas-Pérez, Ulises Rosas, Alejandro Vallejo-Zamora

**Affiliations:** 1 Jardín Botánico, Instituto de Biología, Universidad Nacional Autónoma de México, Tercer Circuito Exterior, S/N, Ciudad Universitaria, Coyoacán 04510, Mexico; esz@ib.unam.mx (E.S.-Z.); urosas@ib.unam.mx (U.R.); vallejo@ib.unam.mx (A.V.-Z.); 2 División Químico Biológicas, Universidad Tecnológica de Tecámac, Carretera Federal México-Pachuca km 37.5, Col. Sierra Hermosa, Tecámac 55740, Mexico; fsalinasp@uttecamac.edu.mx

**Keywords:** *Agave salmiana*, leaf anatomy, leaf anatomical diversification, multivariate analysis, structural variation

## Abstract

*Agave salmiana* Otto ex Salm-Dyck is an endemic Mexican plant distributed from 1230 to 2460 m above sea level, native to the arid zones of central and southern Mexico. It is a traditionally used species, with morphotypes ranging from wild to cultivated, with an ample cultural and management history. The species is important because it generates employment, and its products are used for self-consumption and are marketed as raw materials; however, little is known about its leaf anatomical description or studies that report the variation in its characters in terms of its level of management and its altitudinal gradient. To address this, we collected leaf samples from three localities of the Teotihuacan region in the State of Mexico (Mexico) and obtained anatomical leaf sections; with these, we also obtained thirty-eight parameters to quantitatively describe leaf anatomy. Thus, in this study, the general anatomical description of the leaf of *Agave salmiana* subsp. *salmiana* is presented. Unique leaf characters and others shared with the species of the genus were identified for the leaf of *A*. *salmiana* subsp. *salmiana*. In addition, significant variation was observed when comparing the three sampled localities (78.95%). From the analysis of anatomical characters, abaxial outer periclinal wall length, length of adaxial palisade parenchyma cells, fiber length, surface area of abaxial epidermal cells, width of abaxial palisade parenchyma cells, and total length of parenchyma in adaxial palisade were found to distinguish individuals from the three localities analyzed and the differences are related to management and altitude gradients.

## 1. Introduction

In the Americas, 210 to 273 agave species (*Agave* L., Asparagaceae) have been reported [1,2,3]; 75% of them are present in Mexico, and more than 50% are endemic [4,5,6]. In Mesoamerica, several species of agave have economic relevance. For more than 3500 years, particularly in the regions of Tula, Tulancingo, and Teotihuacan in Mexico, agaves have been used to obtain the fermented drink called pulque [7].

The interaction between agaves and humans in Mesoamerica over thousands of years has increased its diversity, and this interaction is reflected in the 570 common names assigned to 102 variants of agave species [8,9,10,11]. The agave species in Mexico have been used to satisfy and complement a series of basic needs such as food, and have also been used as fiber, fodder, medicines, construction materials, and for the production of alcoholic beverages such as pulque and distillates from aguamiel [9,11,12,13,14].

Given the close relationship between humans and agaves, variants have been detected from which there is comprehensive traditional knowledge regarding agave morphological and ecological variation [15]. This is the case of species belonging to the section *Salmianae*, which includes *A. salmiana* Otto ex Salm-Dyck, a species used mainly for the production of pulque from “aguamiel” (mead), which has the highest number of records and varieties that are part of the traditional agroforestry systems of Mexico [9,11,16]. Management gradients can be determined according to the products that are extracted from the plants (textiles, extraction of mead, extraction of mixiote, etc.), or according to the management of the plants (extensive cultivation, fertilization, pruning, etc. [17]). Likewise, as in other species, a range of management types has been detected in *Agave salmiana*, where it is possible to identify wild and cultivated populations. This management gradients is usually correlated with different genetics and morphologies [9,11].

Morphological studies reported by [18] identified that *Agave salmiana* includes three subspecies: *A. salmiana* subsp. *crassispina* (Trel.) Gentry, *A. salmiana* subsp. *salmiana* Otto ex Salm-Dyck, and *A*. *salmiana* subsp. *tehuacanensis* (Karw. ex Salm-Dyck) García-Mend. *A. salmiana* subsp. *salmiana* has 31 varieties distributed from Tlaxcala to Coahuila [9]. Despite their functional traits and genetic variation, the anatomical differentiation within each of these subspecies (i.e., distinct structural features within the species) is still unknown [11,19,20].

When comparing the anatomy of several organs of agave species, the leaf is the organ with larger intra- and interspecific variation [21,22,23,24,25,26,27]. It is common for plant species to have variations in their functional traits such as structural, genetic, and physiological characteristics that influence their biological success, which has allowed them to overcome the environmental filters of the ecosystem [28]. Variation in these traits would be linked to the processes of response to the climatic conditions of the environment, or to different types and levels of disturbance, management, and domestication [9,11,29].

Vegetative anatomical studies have been demonstrated to be useful in understanding intraspecific variation and its relationship with the environment in several species [10,25,30,31,32]. Although in the genus *Agave*, studies have focused on understanding intra- and interspecific variation, those works have mainly addressed the morphological and genetic variability, as well as their relationship with the management of this phylogenetic resource throughout its history [9,11,13,33,34,35], and yet anatomical approaches have been barely addressed. In this regard, our previous work [25] has reported the variation in the epidermal characters of *A. salmiana* subsp. *salmiana* from ornamentals and cultivated plants from the same three localities of Teotihuacán in the State of Mexico. In order to provide information regarding the leaf anatomy of one of the most important resources for mead extraction and pulque production in Mexico, in this work we describe the detailed leaf anatomy of *Agave salmiana* subsp. *salmiana*, particularly using individuals from one of the most important pulque-producing regions, Teotihuacán in the State of Mexico, Mexico. In addition, we evaluated the quantitative anatomical–foliar variation in this subspecies, comparing plants from three localities and two management gradients: transplanted as ornamentals vs. intensively cultivated for mead extraction, taking together the 14 leaf epidermal characters previously described [25], as well as a new set of 24 characters of the fundamental and vascular tissues of the leaf. Thus, the current work aimed at 1. contributing a detailed descriptive study of leaf anatomy in *Agave salmiana* subsp. *salmiana*; 2. carrying out a comparative analysis of the anatomical characters from three localities of Teotihuacán in the Estado de México to investigate the intraspecific diversity of anatomical–foliar character; and 3. identifying whether this variation is related to environmental conditions (altitude), or to the degree of management intensity to which the plants are subjected in these locations.

## 2. Results

### 2.1. Anatomical Description of the Leaf of Agave salmiana subsp. salmiana

**Figure 1:**
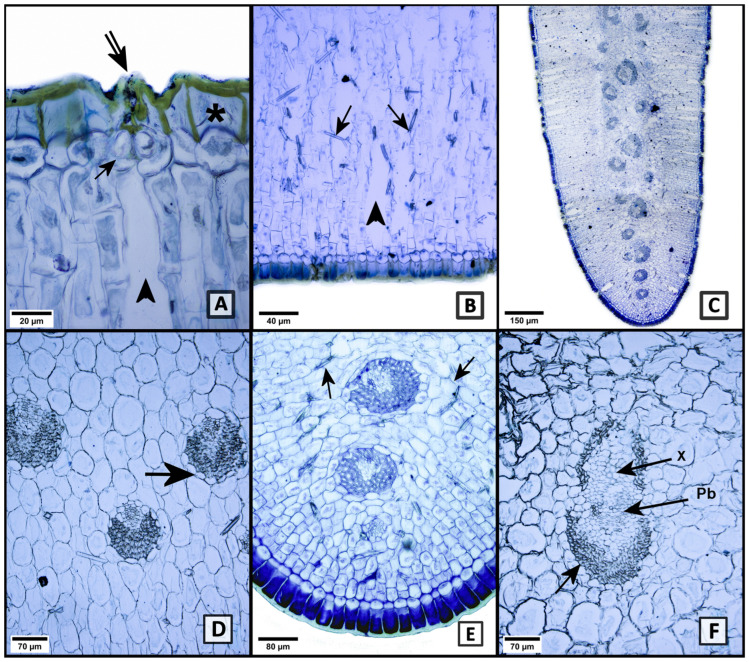
Cross-section of the leaf of *Agave salmiana* subsp. *salmiana*. (**A**) Adaxial epidermis with smooth thick cuticle, a single layer of oblong cells with greatly thickened outer periclinal walls (asterisk). Stomata with guard cells sunken (arrow), but at the level of the rest of the epidermal cells, with prominent external cuticular ridges (double arrow). Elongated substomatal chamber (arrowhead). (**B**) Abaxial epidermis, broad substomatal chamber (arrowhead), and styloid in palisade parenchyma (arrows). (**C**) Margin with reserve and palisade parenchyma, and vascular bundles in a central position. Abaxial left side, adaxial right side**.** (**D**) Small vascular bundles with sclerenchyma sheath outside the phloem and parenchyma sheath around the bundles (arrow). (**E**) Margin. Vascular bundles surrounded by a sclerenchyma sheath. Parenchyma with styloid (arrow). (**F**) Middle vein. Major vascular bundles, sclerenchyma sheath in xylem (X) and phloem (Pb), and parenchyma sheath around the vascular bundle (arrow).

The description of the epidermis in superficial view was previously reported in [25], so in this study, only the epidermis in transverse view is described. The epidermis is unstratified, with oblong cells, the adaxial cells being larger than the abaxial cells; its external periclinal walls are cellulosic and thickened (Figure 1A asterisk). The cuticle is smooth and thick (Figure 1A,B), and at the adaxial end, it is thicker. The stomata are submerged (Figure 1A arrow); however, they are at the same level as the rest of the epidermal cells and without internal cuticular ridges; the external cuticular ridges are very prominent and form a suprastomatic chamber (Figure 1A double arrow); in addition, a very elongated substomatic chamber is observed that involves several layers of mesophyll cells (Figure 1A,B arrowhead).

The mesophyll is formed by palisade parenchyma towards both ends, and towards the center of the lamina, there is reserve parenchyma (also known as spongy parenchyma) (Figure 1C). The palisade parenchyma forms bands of similar thickness at both ends of the leaf; the bands have elongated cells, which are usually rectangular, oriented perpendicular to the lamina surface, and the cells are arranged in rows immediately below the epidermis. These cells are generally twice as long as they are wide, both adaxially and abaxially. The reserve parenchyma contains isodiametric cells of varying sizes.

The vascular tissue is formed by numerous collateral vascular bundles with phloem located towards the leaf surface and xylem to the inner leaf side whose arrangement is opposite; that is, the leaf is isobilateral (Figure 1C). Towards both ends, smaller vascular bundles are located just at the boundary between the palisade parenchyma and the reserve parenchyma (Figure 1D,E). The larger vascular bundles are located in the central part of the mesophyll and are immersed in the reserve parenchyma (Figure 1C,F).

The vascular bundles are arranged in rows, and, according to their area, we categorized them into three different sizes: small (5000–30,000 μm^2^), medium (31,000–50,000 μm^2^), and large (≥51,000 μm^2^). Each vascular bundle contains a sheath of non-septate, cellulose-walled libriform fibers outside the phloem and xylem. This sheath has more layers towards the phloem, particularly in the peripheral vascular bundles (Figure 1D). In addition, a single-layer parenchyma sheath is also observed in each bundle (Figure 1D,F arrow). Both fibers, tracheids and fibro tracheids, are slightly more than 1000 μm in length; however, fibers and tracheids have thicker walls, the latter being the thickest. As cellular contents, calcium oxalate biominerals are observed as styloids immersed in the palisade parenchyma (Figure 1B,E arrows); they are thin and 87 μm long.

### 2.2. Anatomical Variation Between Localities

**Figure 2:**
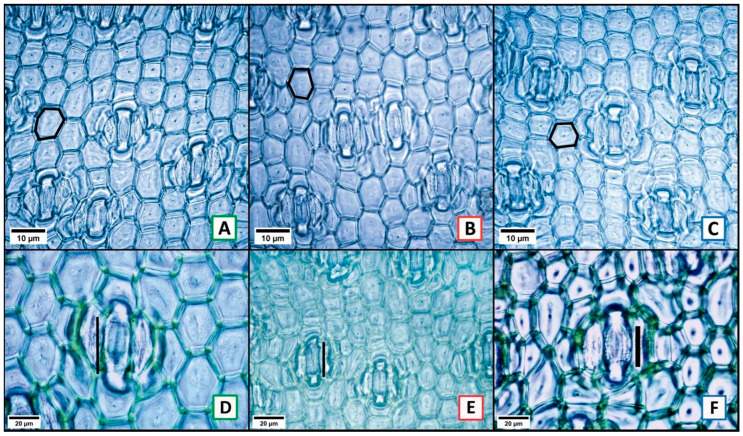
Variable characters among the three locations of *Agave salmiana* subsp. *salmiana*. Front view: surface area of the adaxial epidermal cells (**A**–**C** black circle); length of the abaxial guard cells (**D**–**F**). Letters with a green frame = Tecámac, red = San Martín de las Pirámides, and blue = Teotihuacán.

**Figure 3:**
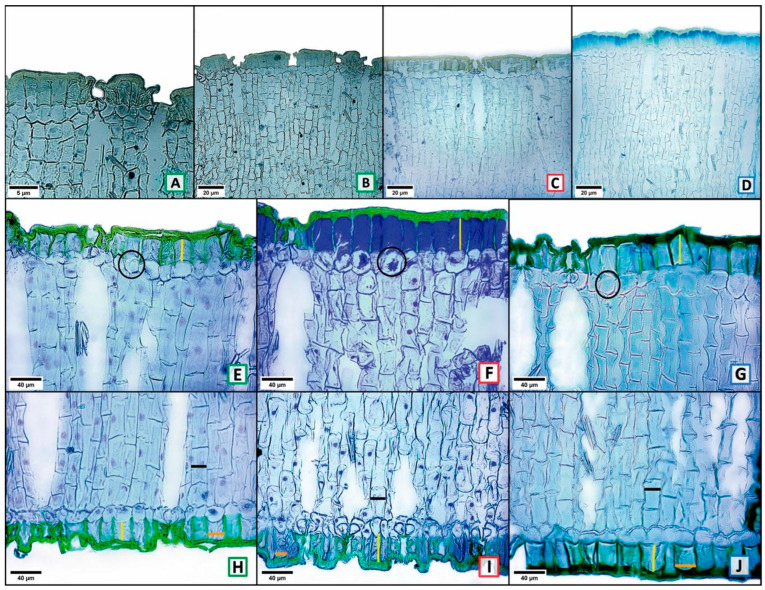
Cross-section: adaxial cuticle width (**A**–**D**); adaxial outer periclinal wall length (**E**–**G** yellow bar); cross-sectional area of the adaxial epidermal cells (**E**–**G** black circle); abaxial outer periclinal wall length (**H**–**J** yellow bar); abaxial outer periclinal wall width (**H**–**J** orange bar); and wide abaxial palisade parenchyma cells (**H**–**J** black bar). Letters with a green frame = Tecámac, red = San Martín de las Pirámides, and blue = Teotihuacán.

**Figure 4:**
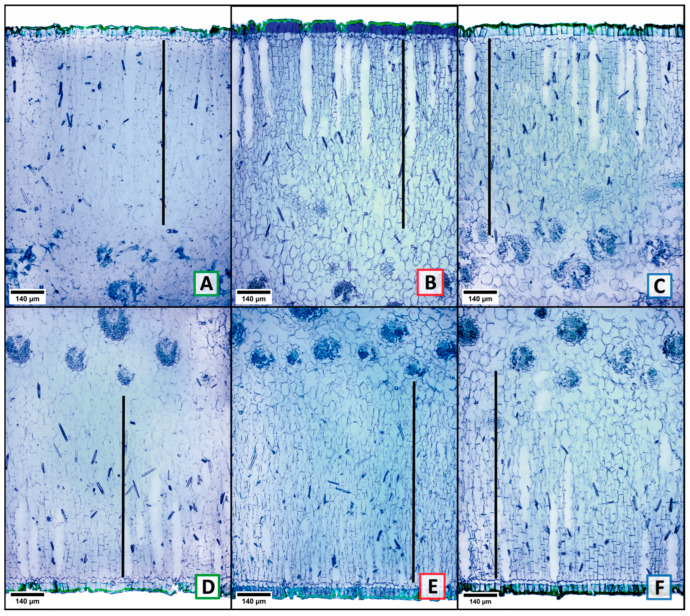
Cross-section: total length of the adaxial palisade parenchyma (**A**–**C** black bars); total length of the abaxial palisade parenchyma (**D**–**F** black bars). Letters with a green frame = Tecámac, red = San Martín de las Pirámides, and blue = Teotihuacán.

From the comparison among the three localities (one-way ANOVA with three levels, one per location), 30 characters (78.95%) were found to show significant variation (*p* ≤ 0.05) in at least one of the three localities (Table 1). The most variable characters among the three locations were 11 (28.95%), and these were the surface area of adaxial epidermal cells (ASCEADA, Figure 2A–C); abaxial guard cell length (LCOABA, Figure 2D–F black bar); adaxial cuticle width (ACADA, Figure 3A–D); cross-sectional area of adaxial epidermal cells (ACEADA, Figure 3E–G); adaxial and abaxial outer periclinal wall length (LPPADA, Figure 3E–G; LPPABA, Figure 3H–J, yellow bars); abaxial outer periclinal wall width (APPABA, Figure 3H–J orange bar); wide abaxial palisade parenchyma cells (ACPABA, Figure 3H–J black bar); total length of parenchyma in adaxial and abaxial palisade (LPADA, Figure 4A–C; LPABA, Figure 4D–F black bars), and fiber length (LFIB). The other 19 characters (50%) showed less variation, splitting the localities only into two groups (Table 1). Only 8 characters (21.05%) had no significant variation among the three localities, and these were adaxial stomatal index (IEADA), width of the abaxial polar cuticular ridge (ARCPABA), adaxial and abaxial polar cuticular space width (AECPADA/AECPABA), fiber width (AFIB), and fibro tracheid length, width, and wall thickness (LFTRA, AFTRA, GPFTRA; Table 1).

**Figure 5:**
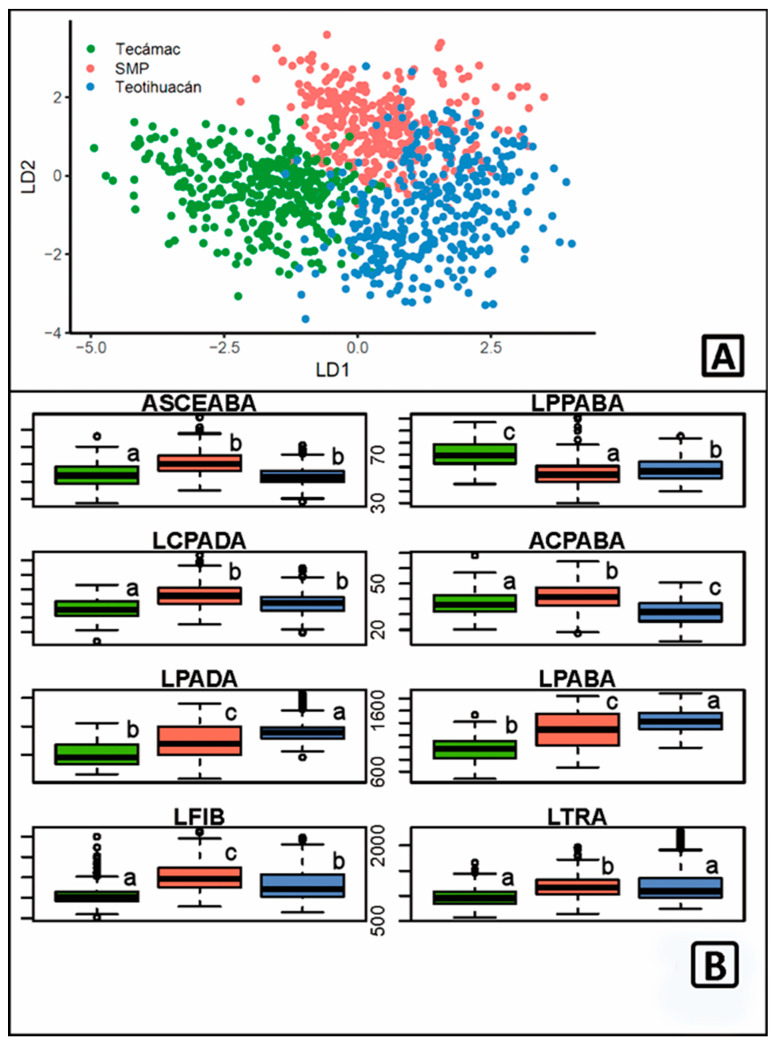
(**A**) Graph of linear discriminant analysis (100% model precision). (**B**) Boxplot for the eight characters with the highest values; the first four characters correspond to the first discriminant (LD1) and the last four to the second discriminant (LD2). Green boxplot = Tecámac, red = San Martín de las Pirámides, and blue = Teotihuacán. Unequal letters indicate different groups in Tukey’s test (*p* < 0.05).

To test whether all together, the anatomical characters can be useful to distinguish between localities, a linear discriminant analysis was performed which showed that the three localities are distinguished with a model accuracy of 100% (Figure 5A). The variables that support this distinction in the first linear discriminant axis are abaxial outer periclinal wall length (LPPABA); length of adaxial palisade parenchyma cells (LCPADA); fiber length (LFIB), and tracheid length (LTRA); while in the second discriminant axis is supported by the characters surface area of abaxial epidermal cells (ASCEABA), width of abaxial palisade parenchyma cells (ACPABA), and total length of parenchyma in adaxial and abaxial palisade (LPADA/LPABA; Table 2). Figure 5B shows the patterns and level of variation in these characters using boxplot plots.

## 3. Discussion

In this study, a detailed description of the leaf anatomy of *A. salmiana* subsp. *salmiana* is presented, as well as the quantitative analysis of 38 anatomical characters to show that they allow us to distinguish the three localities of Teotihuacán agaves (Tecámac, San Martín de las Pirámides, and Teotihuacán). To do this, some of our previously published data on the epidermis was reevaluated [25]. Thus, the current research shows a detailed description of the leaf anatomy, and extends the anatomical analysis and the variation in these characters in *Agave salmiana* subsp. *salmiana*, integrating into the analysis those characters that correspond to the fundamental and vascular tissues, demonstrating the power of integrative quantitative analyses towards the evaluation of variants in *Agave*. Below, we discuss some qualitative and quantitative aspects of this research.

### 3.1. Anatomical Aspects in Asparagaceae

In *A. salmiana* subsp. *salmiana*, a smooth and thick cuticle was observed as in the other *Agave* species that also grow in xerophytic environments [14,36,37,38,39]. It presents a monostratified epidermis as in *Agave fourcroydes* Lem [24], *Agave sisalana* Perrine [14], and in most genera of Asparagaceae [39]. The outer periclinal wall of the epidermal cells in *Agave salmiana* subsp. *salmiana*, although cellulosic, is extremely thickened; however, this condition is not typical of the genus, since there are some species that do not have a thickened wall, such as *A. bracteosa* S. Watson ex Engelm. and *A. ellemeetiana* K. Koch. However, in the other species of *Agave* (*A. sisalana* and *A. striata* Zucc.) and other Asparagaceae such as *Dasylirion longissimum* Lem, *Furcraea macdougalii* Matuda, *Hesperaloe nocturna* Gentry, and *Yucca thompsoniana* Enrejado., the external periclinal wall of the epidermal cells is very thickened. *Agave salmiana* subsp. *salmiana* has sunken stomata on both epidermis, as has been observed in other *Agave* species *(A. sisalana*; [14]. Both species share the presence of rather elongated substomatal chambers. According to [40], along the substomatal chamber, they establish a long and shallow diffusion gradient between the parenchyma and the environment, as well as a high resistance to water loss.

The leaves of *Agave salmiana* subsp. *salmiana* are isobilateral, the mesophyll is formed by palisade parenchyma towards both ends, and towards the center it displays reserve parenchyma. This condition is also present in *A. fourcroydes* [24], *A. sisalana* [14], and *A. striata*, as well as in other Asparagaceae such as *Beschorneria calcícola* García-Mend, *Dasylirion cedrosanum* Treli. [41], *Furcraea macdougallii*, and *Yucca thompsoniana*. In our study, in *A. salmiana* subsp. *salmiana* leaves, the reserve parenchyma shows variation in cell size and shape. Similarly, for *A. bracteosa* and *A. striata*, the reserve parenchyma contains isodiametric cells of different sizes; however, in other species such as *A. fourcroydes*, those cells are irregular [24]. The reserve parenchyma of the photosynthetic organs of succulent species such as Cactaceae, *Aloe*, *Sansevieria*, and *Agave* have large cells with thin walls and large vacuoles containing mucilage [42]. According to the author, these characteristics indicate a tissue specialization that seems to increase the water absorption and storage capacity of the cells, which seems to be an adaptive advantage in *Agave salmiana* subsp. *salmiana* growing in water-deficient environments.

The vascular bundle arrangement in *A. salmiana* subsp. *salmiana* is typical of isobilateral leaves where although each vascular bundle is collateral with phloem located towards the leaf surface and xylem to the inner leaf side, their arrangement is opposite; in other words, the bundles are located with the phloem towards the adaxial and abaxial end, while the xylem poles are located towards the center of the leaf. This arrangement is also observed in *A. fourcroydes* [24] and *A. striata* [14], but it is not exclusive to *Agave* because it has also been observed in other Asparagaceae such as *Dasylirion cedrosanum* [41] and *Furcraea macdougallii.* The vascular bundles are arranged in rows across the width of the leaf, but depending on the region of the leaf (margin or central region), they contain different numbers of peripheral and central bundle layers. In *Agave salmiana* subsp. *salmiana*, each vascular bundle contains two sheaths; the inner sheath consists of fibers that cover the phloem and xylem; however, on the phloem, the number of layers is greater, a condition also characteristic in *A. fourcroydes* [24,43]. The external sheath is parenchymatous and forms a single layer. Some authors have reported the presence of numerous fibers around the vascular bundles in *A. angustifolia* Steud. and *A. tequilana* F. ACWeber [44] and in *Dasylirion cedrosanum* [41]; these authors mention that the walls of these fibers are lignified so their presence provides mechanical strength and rigidity to the leaf. However, in *A. salmiana* subsp. *salmiana,* although the vascular bundles present a fiber sheath, a previous study found through histochemical tests that the cell wall of these fibers is not lignified.

In *Agave salmiana* subsp. *salmiana*, we observed calcium oxalate crystals in the form of styloids immersed in the palisade parenchyma; these are long and thin. Salinas et al. [45] comment that these crystals are commonly present near the substomatal cavity in palisade parenchyma cells with various forms (druses, styloids, or raphides). In *A. americana* L., *A. angustifolia*, *A. atrovirens* Karw. ex Salm-Dyck, and *A. tequilana*, where in addition to styloids, druses and raphides are observed [23,44,45,46,47]. In *Agave sisalana*, rectangular crystals were observed, which are found in larger concentrations among the cells of the palisade parenchyma [14]. Crystals can occur in all plant tissues, and their presence may be associated with the processes of the elimination of excess calcium from the cytosol, as they can act as mechanical support and protection against herbivory [48,49].

The concentration of crystals is higher in agave than in other plants of agricultural importance [23]. In *A. tequilana*, these compounds are the cause of dermatitis in workers of tequila distilleries. The presence of calcium oxalate crystals has been reported as a possible protection mechanism of agave plants against insects and herbivory, as they provide turgor and may be the storage forms of calcium and oxalic acid [23,50].

### 3.2. Variation Between Localities

From these analyses, it can be seen that most of the variable characters among the three localities of *Agave salmiana* subsp. *salmiana* in the Teotihuacán region corresponds to the dermal system of the leaf, as well as to the attributes of the palisade parenchyma, length of fibers, and tracheids.

Given the localities studied, it is presumed that the anatomical variation detected may be the result of the influence of different microenvironmental factors and/or the level of management to which the plants are subjected at each site. Within a management gradient for pulque agaves, Figueredo-Urbina et al. [11] established that the individuals classified as tolerated and transplanted are medium-sized plants with small leaves. In this study, the Tecámac plants have an incipient degree of management through sporadic irrigation and occasional pruning, and are used as ornamentals, so they could fall into the transplanted category, as they are less vigorous and their leaves are also small (1.48 m long). Despite their small size, anatomically, the leaves are distinguished by higher values in the characters that differentiate the localities: the surface area of adaxial epidermal cells, guard cell length, outer periclinal wall width, width of palisade parenchyma cells, cross-sectional area of adaxial epidermal cells, and fiber length.

Mora-López et al. [9] mention that in *Agave*, the pulque variant *Agave salmiana* subsp. *salmiana* with intensive cultivation develops plants of medium to large corpulence and broad leaves, with greater volume of sap used to make pulque, shorter and weaker teeth, and short and fragile apical or terminal spines and thick cuticles, and is classified as “domesticated”. In our study, the plants from San Martín de las Pirámides and Teotihuacán are subjected to intensive cultivation with the pruning of leaves and tillers, irrigation, and frequent supply of organic matter, and they are “capped” and scraped for mead production and therefore receive a higher degree of management. The plants from these localities show lower values in the same anatomical characteristics mentioned for Tecámac. These findings seem to have an inverse trend to that reported by Figueredo-Urbina et al. [11], who mention that cultivated plants with certain management usually show a tendency towards gigantism in the size of their plants and leaves, although less numerous and larger teeth on the margin size.

The shape and size of epidermal cells can show differences between varieties or species [51,52]. In this study, the size of the adaxial epidermal cells, evaluated from their superficial and transversal area, is different in the three localities of *Agave salmiana* subsp. *salmiana*, where the Tecámac plants have the highest values, even though their leaves are shorter. It is observed that there is an inverse relationship between epidermal cell size and altitude since Tecámac is present at a lower altitude (2298.5 m) and its epidermal cells are the largest, while the plants growing in San Martín de las Pirámides and Teotihuacán grow at higher altitudes (2581.5 and 2396, respectively) and have smaller epidermal cells. The size of the epidermal cells could also be inversely related to the lower or higher degree of management of these plants, with larger cells in Tecámac (ornamental) and smaller cells in the remaining two localities (cultivated).

The leaves of *Agave salmiana* are generally amphistomatic and their stomata are sunken [9]. The plants of *Agave salmiana* subsp. *salmiana* from the three studied locations also display stomata in both epidermis as well as *Agave salmiana* subsp. *crassispina,* (personal observation), so it seems to be a genetically determined trait; however, not all agave species have amphistomatic leaves. Several studies in other species have emphasized the relationship of stomata location with different environmental conditions [53]. Amphistomatic leaves are mentioned to be present in the plants whose leaves are uniformly illuminated [54]. Stomatal patterns are widely used in the characterization of plants belonging to different ecotypes, all of which have amphistomatous leaves, and are established as genetically determined characters [55]. Neto and Martins [14] state that in many xerophytic plants, the development of stomata on both sides of the leaf should reduce the limitation of CO_2_ diffusion and uptake, contributing to the increase in the photosynthetic process; so in *Agave salmiana*, the location of stomata seems to be an adaptive advantage. That, together with the supply of water, mineral nutrients, and other abiotic factors, intervene in the structural characteristics of the plant and consequently in its physiology and productivity.

The size and density of stomata have allowed the characterization of varieties growing in different sites, which have been related to variations in drought resistance, amount of shade, NO_2_ content of the air [56,57], and altitude [52,58]. In other investigations, stomatal density showed a positive correlation with altitude [55]. In the present study, it was found that *Agave salmiana* subsp. *salmiana* plants from the three localities are located in an altitudinal range from 2298.5 to 2581.5 m; it was observed that the guard cell length is notoriously longer (45.95–57.00 μm), particularly in the abaxial epidermis, and that character is different among the three localities. Although it is mentioned that the other species have a positive relationship between the guard cell length and altitude, in *Agave salmiana* subsp. *salmiana* of these localities, a negative relationship was observed since Tecámac with a lower altitude has the longest stomata, and San Martín de las Pirámides and Teotihuacán with higher altitudes show the shortest stomata.

It has been observed that the abundance of leaf stomata varies depending on the altitude [59,60,61,62] and with environmental conditions [56]. However, in *Agave salmiana* subsp. *salmiana*, the abundance of stomata did not show a significant variation (*p* > 0.05) in the three localities studied. Nevertheless, in San Martín de las Pirámides, the highest altitude locality (2581.5 m), the plants have a greater abundance of stomata but of small size.

In the other species, a close relationship between stomata abundance and size has been found [57,63]. In addition to altitude and stomata size, stomata abundance in the plants from San Martín de las Pirámides is also associated with greater adaxial cuticle thickness as well as external periclinal wall thickness in both epidermis and greater wall thickness in fibro tracheids. It is known that plants growing at high altitudes, from 2500 onwards, tend to be more xeromorphic, where there is a combined effect of water deficit, high light intensity, extremely low night temperatures, and lower CO_2_ concentration [52], some anatomical trends observed in these plants are thicker cuticle, reduced and scarce stomata located in both epidermis (amphistomatous leaves), and sunken, thick epidermal walls [52,64,65].

Although agaves are adapted to arid and semi-arid climates among other things because they have the acid metabolism of crassulaceae and their leaves are succulent, as in other groups of plants, the size and abundance of stomata are closely related to the environment in terms of efficient gas exchange and water economy [66]. Tecámac plants are managed as ornamentals, have shorter leaves (1.48 m), have larger and more abundant stomata in both epidermis, and have a negative relationship with altitude which is lower (2298.5 m), but also a positive correlation with precipitation which is the highest among the three localities (636 mm). On the other hand, the cultivated plants from San Martín de las Pirámides and Teotihuacán, which grow at higher altitudes (2581.5 and 2396 m, respectively), receive less precipitation (600 and 586 mm), are exposed to hot and dry air, have larger leaves (1.66 and 1.61 m), and therefore a larger leaf surface that favors more evapotranspiration, they have smaller and less abundant stomata to preserve water for longer periods. This same stomatal pattern has been observed in other species where the smaller size and abundance of stomata proved to be an advantage in regulating water transpiration and gas exchange in the photosynthesis process [57,67,68].

Plants respond to environmental variations, particularly water availability, through morphological, anatomical, physiological, and biochemical adjustments that help them cope with such variations [69]. In particular, they adapt to drought stress by developing xeromorphic characteristics such as increased cuticle thickness and enlarged cell walls [69]. For example, leaf cuticular waxes could moderate the effects of drought, and reduce nonstomatal water loss and the diffusion of solutes across the cuticle [42,70,71]; at the same time, cuticular waxes provide natural barriers to the penetration of pathogens [14]. Likewise, the width of the adaxial cuticle and the thickness of the external periclinal wall in both epidermis are considered as barriers against solar radiation and temperature extremes to avoid excessive water loss by evapotranspiration causing water deficit stress [69,72,73]. It has been mentioned that cuticle thickness is related to altitude, particularly in growing species in high mountain xeric environments, where their leaves have a cuticle 3.2 to 4.3 μm thick [58]. In *Agave salmiana* subsp. *salmiana*, the thickness of the adaxial cuticle is significantly different in the three localities; however, the abaxial cuticle is similar for Tecámac and Teotihuacán. Nevertheless, the plants from San Martín de las Pirámides with long leaves (1.66 m) and an average annual temperature range between 10 and 30 °C have the thickest cuticle for both epidermis, a condition that seems to be positively related to their altitude (2581.5 m). In several agro-ecological systems where *Agaves* are cultivated, it is common to find water deficit that can be the result of low rainfall, low water retention capacity in the soil, excessive salinity, and extreme cold or hot temperatures, among other factors [74]; hence, the presence of a thick cuticle may represent an adaptive advantage in these plants.

Given its greater cuticle thickness and epidermal walls, it is proposed that in particular, the plants of San Martín de las Pirámides be used for the extraction of “mesiote or mixiote” supporting what was previously suggested by Mora-López et al. [9] for *Agave salmiana* subsp. *salmiana*. On the other hand, it is necessary to select appropriate ecotypes for the preservation of the diversity of the local flora and to optimize reforestation programs. Therefore, it is proposed that *Agave salmiana* cultivated varieties of economic interest, with thick cuticles and epidermal walls, are used for agricultural production because they have greater resistance or tolerance to water deficit; this knowledge could provide a basis for efficient agronomic production and/or the utilization of this resource.

The development of a compact palisade parenchyma is associated with xeromorphic plants present in high-altitude environments with strong solar radiation [69,75]. *Agave salmiana* subsp. *salmiana* also develops a compact palisade parenchyma at both sides of the leaf and it is a common trait of the plants at all three locations studied. However, the total length of the palisade parenchyma on both sides is different at these locations.

Teotihuacán plants are distributed at altitudes of 2396 m, in dry environments that receive less annual precipitation (586 mm), and are highly exposed to light and have large leaves (1.61 m long by 32.8 cm wide). These leaves show the highest values for the total palisade parenchyma length at both the abaxial and adaxial sides. This same trend was observed in other species where it is proposed that greater leaf and palisade parenchyma thickness may optimize plant survival and growth by improving water relations and providing greater protection for internal tissues in sites with high aridity conditions [58,69,76,77]. On the other hand, it has been observed that there is a positive relationship between the thickness of the palisade parenchyma and the level of solar radiation or light exposure as one ascends [62,78]. This same trend was observed in the plants of Teotihuacán.

The plants of San Martín de las Pirámides, even though they also grow in sites of higher altitude and reduced precipitation, have a smaller total thickness of the palisade parenchyma, which indicates a negative relationship; however, the presence of other structures such as a smaller size of the stomata and greater cuticle thickness may compensate for the water economy and the protection of the internal tissues. On the other hand, its leaves, although long, are the narrowest (1.66 m long by 30.6 cm wide), and there could be a positive correlation between leaf width and the presence of a less extended photosynthetic parenchyma, which may be determining the accumulation of sugars derived from photosynthesis [73], and therefore the quality of “aguamiel” from these plants. However, these aspects require further studies.

Castillo et al. [79] analyzed the variation in some attributes of fibers from plants grown in five different localities of *Agave lechuguilla* Torr., mentioning that the fibers had a diameter of 293 μm, while the fibers of *A. salmiana* subsp. *salmiana* on average had a significantly smaller diameter (22.60 μm). In our study, no significant variation in fiber width (*p* > 0.05) was observed among individuals from the three locations evaluated. Nevertheless, the plants from San Martín de las Pirámides with narrow leaves have fibers with lower wall thickness, which suggests that there is a positive relationship between these parameters.

Several authors suggest that there is a relationship between fiber length and altitude [52,62,80,81]. Fiber length is a significantly different character (*p* > 0.05) among the three populations of *Agave salmiana* subsp. *salmiana*, where a negative relationship with altitude was observed, i.e., at lower altitudes as in Tecámac (2298.5 m), the fibers were longer, while in San Martín de las Pirámides with higher altitude (2581.5) the fibers were shorter; these results coincide with those reported for other species [52,62]. It is suggested that the agaves cultivated in this locality are preferably selected for the elaboration of textiles of soft consistency, or for the construction of indigenous huts, while those from Tecámac and Teotihuacán have long and thicker-walled fibers, which makes them comparatively more resistant to be used for other purposes. These results confirm the findings of Reyes-Agüero et al., [10] who mention that “The longest and highest quality fibers are present in *Agave salmiana*”.

On the other hand, the anatomical variation in fibers in *Agave salmiana* subsp. *salmiana* is congruent with the hypothesis that a large amount of sclerenchyma (long, thick-walled fibers) provides better mechanical support and protection against herbivores in the leaf [82,83]. Coley et al. [84] mentioned that sclerenchyma production increases with harsher environmental stress. In this subspecies, particularly the plants from the Teotihuacán locality have long, wide, thick-walled fibers as compared to the other localities, and Teotihuacán is the site that receives the least annual rainfall (586 mm); meanwhile, Castillo et al. [79] also mention that plants growing in environments with lower rainfall tend to show larger fiber diameters and higher tensile strength. Likewise, the presence of lignin in the cell walls of the fibers is essential to provide mechanical support and resistance to the tissues that display them [40]; the particular case of the fibers in *A. lechuguilla* did not confirm the nature of the cell wall [79]. However, in *A. salmiana* subsp. *salmiana*, in previous studies, based on histochemical tests, it was confirmed that these fibers were cellulosic. It has been reported that the most common uses of *Agave salmiana* leaves are the use of its fibers for hut construction, the production of ixtle, and the manufacture of ropes or textiles [2,10,85,86,87]. Likewise, here we propose that the leaves of *A. salmiana* subsp. *salmiana* from Tecámac and Teotihuacán are better used for these purposes.

Locosselli and Ceccantini [57] studied the dimensions of the tracheids and their relationship with the size of the stomata in populations of *Podocarpus lambertii* Klotzsch ex Endl. (Podocarpaceae), which grow in two different sites. Their results show that the dimensions of the tracheids vary between these sites. In this study, certain trends could be observed; in individuals from hot and dry areas in northeastern Brazil, tracheids with smaller diameter pits, shorter cells, and thicker cell walls were observed along with smaller stomata [57]. In relation to *Agave salmiana* subsp. *salmiana*, Teotihuacán is the driest locality, with the least precipitation (586 mm) and an average annual temperature range of 6 °C to 31 °C; these plants showed the highest values in terms of the length, width, and wall thickness of the tracheids in their leaves, associated with relatively small stomata, so only the wall thickness and the presence of small stomata coincide with the trend observed in the hot and dry conditions where some populations of *Podocarpus lambertii* grow [57]. On the other hand, in the plants of San Martin de las Piramides, present in the locality of higher altitude (2581.5 m) and intermediate precipitation (600 mm), shorter tracheids and small stomata were found. The presence of these attributes could make the use of water more efficient under drought conditions and could mean an advantage for the plants growing in these localities.

## 4. Material and Methods

This study was conducted at the Plant Anatomy Laboratory of the Botanical Garden, Institute of Biology, National Autonomous University of Mexico. We worked with the same materials used in our previous study on agave epidermis [25], where three localities of Teotihuacán, located in the northwest of the State of Mexico (San Martín de las Pirámides, Tecámac, and Teotihuacán), were evaluated (Table 3). These localities were chosen because in the State of Mexico, as well as in Hidalgo and Tlaxcala, the traditional uses of agave are preserved, maintaining the richness of its variants, in addition to being one of the areas with the largest production of mead for pulque and other beverage manufacturing [9,10,11,13]. In addition, these plants display a gradient of domestication attributes [9,14], meaning that those from Tecámac constitute ornamental plants without any agronomic management, while those from the two remaining localities (Teotihuacán and San Martín de las Pirámides) are plants with greater management intensity (including fertilization, pruning to obtain leaves, capping, scraping to obtain mead, etc).

The middle part of the fresh and healthy blade leaf was selected in relation to the total length of the leaves of plants 6 to 8 years old. Samples were collected from 15 individuals, 5 from each of the 3 localities, and 2 sections were taken from each individual, for a total of 30 samples. A total of 15 of those samples were used to obtain transverse sections to analyze cuticle width, the cross-sectional area of epidermal cells, the length and width of the external periclinal wall, the length and width of palisade parenchyma cells, the total length of palisade parenchyma, and the length of styloids. The other 15 samples were collected to obtain dissociated tissues that allowed the analysis of the length, width, and thickness of the xylem elements (fibers, tracheids, and fibro tracheids). The samples were fixed in the field with FAA (Formaldehyde–Alcohol–Acetic Acid) and preserved in the fixative solution for 7 days.

It was necessary to soften the tissue with 10% ethylenediamine for 20 days because agave leaves have tissue with abundant hard and resistant cellulosic fibers that are immersed within the soft tissue. Subsequently, the samples were rinsed with tap water for 20 min and kept in water for 48 h. All the samples were dehydrated with a gradual series of 35, 50, 60, 70, 85, 95, and 100% tertiary butyl alcohol (ATB) for 24 h in each.

The samples were then infiltrated and embedded according to conventional histological techniques with paraffin (58–60 °C). Histological sections of 15 µm were obtained using a rotating microtome (American Optical, model 820). They were then stained with acid toluidine blue (pH 3.6 and borax) for 30 min and finally mounted as histological preparations with synthetic resin [88].

### 4.1. Processing for Dissociates

The 15 selected samples were kept in 70% ethyl alcohol prior to processing. For each sample, both the adaxial and abaxial epidermis were removed by making paradermal cuts, leaving only the middle part of the lamina where the vascular bundles are located. These samples were cut longitudinally, parallel to the same direction of the vascular bundles and fibers, which facilitated contact with Jeffrey’s solution, allowing the tissue maceration and the separation of fibers, fibro tracheids, and tracheids. The samples were kept for 2 h until the vascular elements were separated. Then, they were rinsed with distilled water for 10–15 min, after which a small tissue sample was placed on a slide and mounted as temporary preparations with glycerinated gelatin [89].

The slides were observed using brightfield, phase contrast, and polarization microscopy with a Carl Zeiss-Axioskop photomicroscope and a Rising View camera (U3ISPM16000KPA, 16MP 1/2.33″ PANASONIC CMOS). The linear and area measurements were performed with the ImageJ v.1.48 program [89].

### 4.2. Statistic Analysis

In order to perform an integral analysis of leaf anatomy, 38 quantitative characters were evaluated for each of the 5 individuals of *Agave salmiana* subsp. *salmiana* from each of the 3 locations (Table 4), from which 14 are the epidermal characters that we previously reported [25], and the rest are published here for the first time. A total of 20 measurements for each one of the 38 characters were taken from the dermal, fundamental, and vascular tissues (760 measurements) from 5 individuals (3800), from each location, which made a total of 11,400 total measurements in the three locations. The normality and homogeneity of variance tests were applied to the data. In order to compare the variation in these anatomical parameters from the three localities, we performed a one-way analysis of variance (ANOVA), having locality as a factor (3 levels) and their corresponding contrast of means using the Tukey test, at a significance level of 5% (*p* ≤ 0.05). For all 38 parameters evaluated, the mean values and standard deviations are reported (Table 4).

With these 38 characters, we performed a linear discriminant analysis to observe whether the three populations can be distinguished and which characters have the greatest weight in this distinction. To do this, the leaf data of the five individuals from each locality were combined, and the character data were standardized prior to the linear discriminant analysis. All the statistical analyses were performed with R version 3.6.2 [90], particularly using the agricolae package version 1.3-3 for the ANOVAs.

## 5. Conclusions

Although only the northwestern region of the State of Mexico was studied, this is the first work that presents in detail the anatomical description of the leaf of *Agave salmiana* subsp. *salmiana*. Six anatomical–foliar characters (abaxial outer periclinal wall length, length of adaxial palisade parenchyma cells, fiber length, surface area of abaxial epidermal cells, width of abaxial palisade parenchyma cells, and total length of parenchyma in adaxial palisade) contribute significantly in distinguishing *Agave salmiana* subsp. *salmiana* populations from the region of Teotihuacan in the State of Mexico. The close relationship that this species has had with man in certain populations has led to different levels of humanization, and this seems to be inducing structural changes in its leaves, such that the plants of Tecámac without agronomic management and lower altitude show some anatomical characteristics with higher values, and in those plants managed under intensive cultivation in San Martín de las Pirámides y Teotihuacán, with a higher level of humanization and higher altitude, more than 30% of the analyzed characters are similar and show lower values in significantly distinctive characters.

It is convenient to explore the anatomical–foliar traits of this same subspecies in other mead-producing localities and in the other two subspecies of *Agave salmiana* in order to elucidate which characteristics are genetically determined and whether there are similar patterns of variation within those subspecies. Our study contributes to the knowledge of the structural diversity of this resource towards the understanding of the processes of change that conserve and diversify resources through their interaction with humans and with the environment. Finally, it is important to mention that the analysis of the anatomy and its corresponding variation is essential to contribute to the knowledge that allows the design of strategies for the better utilization, use, and sustainable management of *A. salmiana*.

## Figures and Tables

**Table 1:** Mean ± one standard deviation of the anatomical parameters of *A. salmiana* subsp. *salmiana* among the three localities. Unequal letters indicate different groups in Tukey’s test (*p* < 0.05). * lowest value, ** highest value. The meaning of the acronyms can be found in the material and methods section.

Character	Tecámac	San Martín	Teotihuacán
IEADA	* 8.30 ± 1.38 a	** 8.72 ± 1.14 a	8.43 ± 1.35 a
IEABA	* 7.20 ± 1.23 b	** 8.09 ± 1.20 a	7.96 ± 1.36 a
LCOADA	** 55.50 ± 8.98 a	* 46.43 ± 5.15 b	47.64 ± 5.85 b
LCOABA	** 57.00 ± 6.31 a	* 45.95 ± 4.49 c	51.13 ± 5.82 b
GPAADA	** 6.22 ± 1.41 a	5.60 ± 1.15 b	* 5.52 ± 1.39 b
GPAABA	** 6.10 ± 1.14 a	5.43 ± 1.15 b	* 5.36 ± 1.12 b
ABCLADA	** 39.32 ± 8.45 a	38.00 ± 8.29 a	* 34.78 ± 6.60 b
ABCLABA	** 36.51 ± 6.98 a	* 33.23 ± 5.85 b	33.88 ± 5.69 b
ARCPADA	* 9.23 ± 2.57 b	** 10.78 ± 2.62 a	9.46 ± 2.52 b
ARCPABA	* 9.09 ± 2.58 b	9.36 ± 2.29 ab	** 10.04 ± 3.01 a
AECPADA	** 40.73 ± 5.98 a	38.82 ± 5.88 ab	* 37.77 ± 8.11 b
AECPABA	** 37.99 ± 6.23 a	* 36.30 ± 7.25 a	37.08 ± 7.39 a
ASCEADA	** 4178.57 ± 714.66 a	3735.87 ± 571.12 b	* 3357.84 ± 568.52 c
ASCEABA	** 4174.01 ± 638.24 a	3368.38 ± 919.57 b	* 3272.91 ± 484.79 b
LEST	** 98.55 ± 22.18 a	83.94 ± 17.63 b	* 82.30 ± 24.25 b
ACADA	19.51 ± 3.56 b	** 22.33 ± 3.96 a	* 18.14 ± 4.61 c
ACABA	18.39 ± 3.55 b	** 22.06 ± 3.90 a	* 18.35 ± 5.67 b
LPPADA	* 52.84 ± 10.82 c	** 75.18 ± 11.35 a	65.22 ± 12.31 b
LPPABA	* 51.33 ± 9.32 c	** 71.25 ± 10.04 a	55.87 ± 8.62 b
APPADA	** 45.21 ± 12.75 a	* 39.77 ± 7.05 b	41.09 ± 8.62 b
APPABA	** 45.26 ± 10.83 a	41.57 ± 6.88 b	* 38.53 ± 8.53 c
LCPADA	** 91.77 ± 15.26 a	* 70.68 ± 14.03 b	75.35 ± 14.92 b
LCPABA	** 95.62 ± 19.22 a	* 74.18 ± 14.25 b	75.11 ± 19.59 b
ACPADA	** 39.97 ± 8.54 a	38.32 ± 6.51 a	* 30.07 ± 9.34 b
ACPABA	** 42.44 ± 7.65 a	39.14 ± 8.22 b	* 29.37 ± 9.05 c
ACEADA	** 2300.20 ± 945.31 a	2010.72 ± 811.66 b	* 1644.14± 567.67 c
ACEABA	1563.34 ± 458.60 a	** 1563.84 ± 490.13 a	* 1189.37 ± 388.78 b
LPADA	1235.26 ± 206.80 b	* 989.01 ± 183.69 c	** 1397.81 ± 94.73 a
LPABA	1252.69 ± 237.70 b	* 996.99 ± 162.20 c	** 1461.41 ± 139.28 a
LFIB	** 1585.85 ± 298.88 a	*1038.30 ± 159.42 c	1311.62 ± 400.81 b
AFIB	22.55 ± 7.10 ab	* 21.38 ± 4.70 b	** 23.86 ± 6.69 a
GPFIB	** 5.93 ± 1.15 a	* 5.19 ± 1.05 b	5.74 ± 1.92 a
LTRA	1212.66 ± 224.43 a	* 952.22± 147.99 b	** 1220.11 ± 378.13 a
ATRA	* 30.24 ± 4.68 b	30.76 ± 4.37 b	** 33.79 ± 13.67 a
GPTRA	* 4.90 ± 1.16 b	5.28 ± 1.18 b	** 6.38 ± 2.82 a
LFTRA	* 1064.76 ± 458.89 a	1104.44 ± 373.92 a	** 1202.64 ± 675.71 a
AFTRA	11.51 ± 2.69 a	* 11.08 ± 28.18 a	** 12.02 ± 3.78 a
GPFTRA	* 2.60 ± 0.74 b	** 2.90 ± 0.73 a	2.84 ± 0.86 ab

**Table 2:** Highest linear discriminant coefficients. Value in parentheses = capture of the linear discriminant. Model accuracy = 100%.

LD1 (0.658)		LD2 (0.342)	
LPPABA	−0.70359314	ASCEABA	−0.64819398
LCPADA	0.54619874	ACPABA	−0.43531551
LFIB	0.36317273	LPADA	0.64223283
LTRA	0.48391477	LPABA	0.59032855

**Table 3:** General data of the samples of *Agave salmiana* subsp. *salmiana* from Teotihuacan, State of Mexico (modified of [25]).

Locality	Altitude (masl)	Age of the Plant (years)	Blade Length (m)	Blade Width (cm)	Annual Temperature Range (°C)	Annual Precipitation (mm)
Tecámac	2238–2359	6–8	1.48 ± 0.22	32.95 ± 4.01	6–31	636
San Martín de las Pirámides	2238–2925	8	1.66 ± 0.05	30.6 ± 2.40	10–30	600
Teotihuacán	2257–2535	8	1.61 ± 0.02	32.8 ± 3.04	6–31	586

**Table 4:** List of characters and their acronyms.

No.	Characters	Acronym
1	Adaxial stomatal index	IEADA
2	Abaxial stomatal index	IEABA
3	Adaxial guard cell length	LCOADA
4	Abaxial guard cell length	LCOABA
5	Adaxial anticline wall thickness	GPAADA
6	Abaxial anticline wall thickness	GPAABA
7	Width of adaxial lateral cuticular border	ABCLADA
8	Width of abaxial lateral cuticular border	ABCLABA
9	Width of adaxial polar cuticular ridge	ARCPADA
10	Width of abaxial polar cuticular ridge	ARCPABA
11	Adaxial polar cuticular space width	AECPADA
12	Abaxial polar cuticular space width	AECPABA
13	Surface area of adaxial epidermal cells	ASCEADA
14	Surface area of abaxial epidermal cells	ASCEABA
15	Styloid length	LEST
16	Adaxial cuticle width	ACADA
17	Abaxial cuticle width	ACABA
18	Adaxial outer periclinal wall length	LPPADA
19	Abaxial outer periclinal wall length	LPPABA
20	Adaxial outer periclinal wall width	APPADA
21	Abaxial outer periclinal wall width	APPABA
22	Length of adaxial palisade parenchyma cells	LCPADA
23	Length of abaxial palisade parenchyma cells	LCPABA
24	Width of adaxial palisade parenchyma cells	ACPADA
25	Width of abaxial palisade parenchyma cells	ACPABA
26	Cross-sectional area of adaxial epidermal cells	ACEADA
27	Cross-sectional area of abaxial epidermal cells	ACEABA
28	Total length of parenchyma in adaxial palisade	LPADA
29	Total length of parenchyma in abaxial palisade	LPABA
30	Fiber length	LFIB
31	Fiber width	AFIB
32	Fiber wall thickness	GPFIB
33	Tracheid length	LTRA
34	Tracheid width	ATRA
35	Tracheid wall thickness	GPTRA
36	Fibro tracheid length	LFTRA
37	Fibro tracheid width	AFTRA
38	Fibro tracheid wall thickness	GPFTRA

## Data Availability

The data presented in this study are available on request from the corresponding author.
